# Application of Biochar-Based Materials for Effective Pollutant Removal in Wastewater Treatment

**DOI:** 10.3390/nano14231933

**Published:** 2024-11-30

**Authors:** Meiyao Han, Ziyang Liu, Shiyue Huang, Huanxing Zhang, Huilin Yang, Yuan Liu, Ke Zhang, Yusheng Zeng

**Affiliations:** 1 College of Civil Engineering, Sichuan Agricultural University, Dujiangyan 611830, China; hanmeiy@stu.sicau.edu.cn (M.H.); liuziyang66@stu.sicau.edu.cn (Z.L.); huangsy@stu.sicau.edu.cn (S.H.); yanghuilin@stu.sicau.edu.cn (H.Y.); zhangke@sicau.edu.cn (K.Z.); 2 Luoyang Petrochemical Engineering Design Co., Ltd., Luoyang 471003, China; zhanghuan_xing@163.com; 3 Chengdu Tiantou Industry Co., Ltd., Chengdu 610000, China; 306liuyuan@163.com

**Keywords:** biochar, catalyst, advanced oxidation processes, wastewater treatment

## Abstract

With the growth of the global population and the acceleration of industrialization, the problem of water pollution has become increasingly serious, posing a major threat to the ecosystem and human health. Traditional water treatment technologies make it difficult to cope with complex pollution, so the scientific community is actively exploring new and efficient treatment methods. Biochar (BC), as a low-cost, green carbon-based material, exhibits good adsorption and catalytic properties in water treatment due to its porous structure and abundant active functional groups. However, BC’s pure adsorption or catalytic capacity is limited, and researchers have dramatically enhanced its performance through modification means, such as loading metals or heteroatoms. In this paper, we systematically review the recent applications of BC and its modified materials for water treatment in adsorption, Fenton-like, electrocatalytic, photocatalytic, and sonocatalytic systems, and discuss their adsorption/catalytic mechanisms. However, most of the research in this field is at the laboratory simulation stage and still needs much improvement before it can be applied in large-scale wastewater treatment. This review improves the understanding of the pollutant adsorption/catalytic properties and mechanisms of BC-based materials, analyzes the limitations of the current studies, and investigates future directions.

## 1. Introduction

Water pollution is a critical global issue, threatening ecosystems and human health [1]. Rapid population growth, industrialization, and urbanization have led to significant pollutant discharges from industrial, agricultural, and urban sources. These pollutants often contain high levels of nitrogen and phosphorus, causing eutrophication, heavy metals like lead and mercury, and organic contaminants such as pesticides and drug residues. This contamination threatens drinking water safety and sustainable water use [2,3]. Conventional water treatment methods are increasingly inadequate, prompting researchers to develop more efficient and environmentally friendly solutions.

Biochar (BC) is a carbon-based material produced through the pyrolysis of biomass, which includes agricultural residues (like straw and branches), municipal sludge, food waste, and other organic materials. These feedstocks are cost-effective and contribute to the reduction of environmental pollution [4]. Common production methods include pyrolysis, hydrothermal treatment, and gasification [5,6,7]. The physicochemical properties of BC vary based on the precursor material and preparation conditions [8]. The most commonly reported physical properties include structure, porosity, pore size distribution, total pore, specific surface area, and adsorption capacity [9,10]. Meanwhile, its chemical properties are typically characterized by moisture content, ash, volatile matter, fixed carbon, carbon content, functional groups, aromaticity, and degree of carbonization [9,11]. The unique physicochemical properties of BC enable its effective use in various applications. In agriculture, BC serves as a slow-release fertilizer that improves soil quality and promotes crop growth [12,13]. In environmental applications, it is employed to control pollutant migration and reduce soil and water contamination [14,15]. In water treatment, BC demonstrates excellent adsorption and catalytic properties due to its large surface area, porous structure, and surface-active functional groups [16,17]. Its ability to remove pollutants stems from its porous design and the presence of oxygen-containing groups (e.g., carboxyl, hydroxyl, carbonyl) that enhance chemical adsorption. However, its performance can be limited to complex pollutants. Researchers have addressed this by modifying BC to improve its pore structure, increase surface area, and enhance activity through the addition of metals and heteroatoms [18,19].

Compared to traditional adsorption processes, advanced oxidation processes (AOPs) are increasingly recognized for their ability to mineralize resistant organic pollutants into harmless by-products like water and carbon dioxide [20]. These processes generate highly reactive species, such as sulfate (SO_4_^•−^) and hydroxyl radicals (HO^•^), which effectively degrade pollutants. However, reliance on metal catalysts can lead to leaching and by-product formation, posing environmental risks [17,21]. Therefore, eco-friendly alternatives like BC have gained traction, as they prevent metal leaching and demonstrate excellent catalytic properties [22]. Studies show that persistent free radicals (PFRs) and oxygenated functional groups (OFGs) in BC activate oxidants, enhancing pollutant degradation [23,24]. Yet, pure BC’s catalytic capacity is limited, prompting modifications through techniques like ball milling and the addition of metals to improve performance [25,26,27]. Modified BC excels in water treatment and is also effective in air pollution control and soil remediation [14].

The pollutant removal performance of BC-based materials depends on their physicochemical properties, the type of pollutant, and treatment conditions. The physicochemical properties of the BC can be modified through activation (acid-base), ball milling, or the addition of metals and heteroatoms [7,25,26,28]. Among the treatment conditions, the amount of BC used is a key factor affecting pollutant removal. More BC catalysts provide additional active sites, enhancing adsorption and promoting the generation of reactive oxygen species (ROS), which improves pollutant degradation efficiency [29,30,31]. However, excessive amounts of BC catalysts may lead to the self-quenching of free radicals [32]. Elevated temperatures enhance pollutant adsorption and help reactant molecules overcome activation energy barriers, facilitating ROS formation [33,34]. However, high temperatures may be uneconomical, so selecting the optimal temperature is crucial. The effect of solution pH on pollutant removal depends on the properties of the substances involved in different degradation systems and the characteristics of the pollutant [35,36]. Additionally, the adsorption of pollutants can be hindered by competing anions (e.g., NO_3_^–^, SO_4_^2–^, PO_4_^3^⁻), which may affect the catalytic process [37,38,39]. To tackle growing wastewater treatment challenges, there is a global push for sustainable materials. BC, a low-cost carbon-based option, has gained attention, though an efficient industrial-scale application is still lacking. This paper reviews recent advancements in BC for water treatment, highlighting its precursor characteristics and their impact on BC’s structure and performance. It discusses BC applications across various treatment technologies, including adsorption systems, Fenton-like systems, electrocatalytic systems, photocatalytic systems, and sonocatalytic systems. The review explores the adsorption and catalytic mechanisms of different BC types and identifies research gaps and future directions, aiming to support the development and wider application of BC in wastewater treatment.

## 2. Production and Structural Characteristics of Biochar

### 2.1. Composition of Biochar

#### 2.1.1. Carbon Sources of Biochar

**Figure 1:**
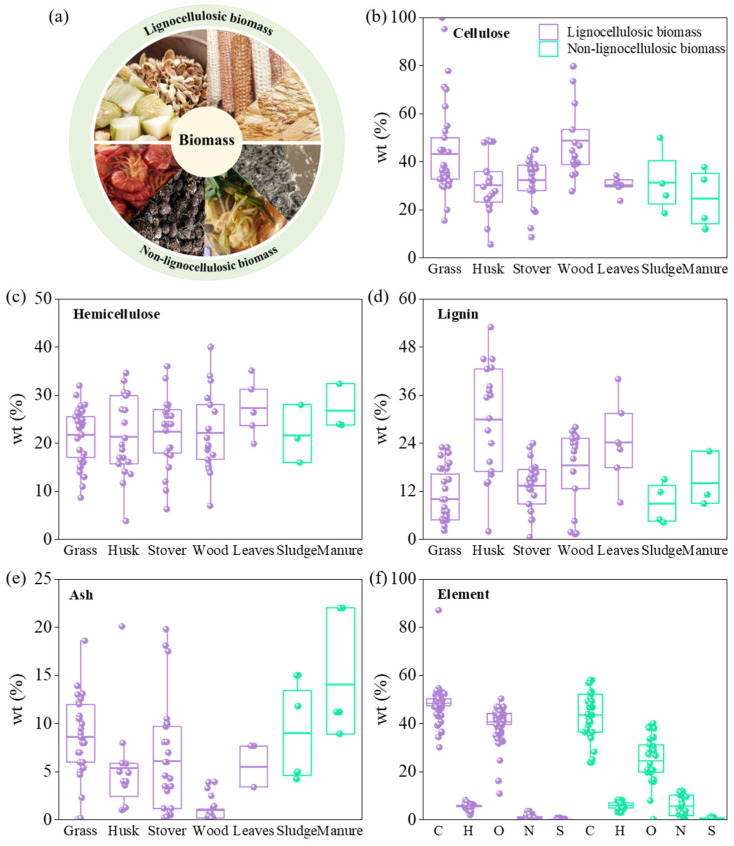
(**a**) Biomass classification. (**b**) Cellulose, (**c**) hemicellulose, (**d**) lignin, and (**e**) ash mass ratios for different types of biomass, and (**f**) average elemental mass ratios for both types of biomass. (Data from the Phyllis2 database).

BC can be derived from various carbon sources, typically categorized into lignocellulosic and non-lignocellulosic biomass. Lignocellulosic biomass, primarily sourced from plants, includes materials rich in cellulose, such as wood, leaf litter, fruit hulls, and straw. This biomass consists of cellulose (25–50 wt%), hemicellulose (15–40 wt%), and lignin (10–40 wt%), along with minor components like structural proteins, lipids, and ash. Non-lignocellulosic biomass encompasses a broader range of materials, including animal and microbial sources, as well as certain plant derivatives [40,41]. Minakshi et al. studied industrial hemp from Western Australia, which consists of two parts: the hemp wood core (65%) and the hemp blast (35%). The wood core contains 40–48% cellulose, 18–24% hemicellulose, and 21–23% lignin, while the embryo contains 57–77% cellulose and 9–14% hemicellulose, which were consistent with the compositional properties of lignocellulosic biomass [42]. Examples of non-lignocellulosic biomass include municipal sludge, food waste, livestock manure, algae, and yeast residues. Figure 1a illustrates the common types of both lignocellulosic and non-lignocellulosic biomass.

#### 2.1.2. Elemental Composition of Biomass

Elemental composition is a key characteristic of carbon materials. Figure 1b–f shows the composition of various biomass types, including grasses (switchgrass, miscanthus, clover), peels and shells (olive husk, almond hulls, coconut husk), straws (corn stover, winter rape straw, rye straw), woods (beech, oak, cherry), leaf litter (olive, maple, palm leaves), sludge (from onion oil and sewage), and manure (from cows, sheep, and horses). These biomass types primarily consist of carbon (C) and oxygen (O), with varying amounts of nitrogen (N) and sulfur (S). The higher carbon content in lignocellulosic biomass is due to its significant cellulose, hemicellulose, and lignin (Figure 1b–e), while hemicellulose tends to decompose completely at elevated temperatures (∼900 °C) due to its low degree of polymerization. Some lignin precursors are rich in specific heteroatoms. For example, Minakshi et al. found that honeydew melon contains high levels of potassium, chloride, and phosphorus. During chemical activation, water-soluble KCl salts may react with HDP under heat-treatment conditions, releasing chloride as a gas and causing potassium to melt into the solid carbon, resulting in the formation of porous graphitic carbon [43]. In contrast, non-lignocellulosic biomass typically contains higher levels of miscellaneous elements such as N, P, S, and metals (Figure 1f). This is likely due to its higher protein and lipid content [40], which incorporates these elements into the structural units. Consequently, non-lignocellulosic biomass, rich in nitrogen, sulfur, and phosphorus, is often utilized to enhance soil quality [12]. As shown in Figure 1d, non-lignocellulosic biomass also tends to have higher ash content, attributed to the presence of elements like P, Ca, K, Mg, Fe, Si, and Na.

### 2.2. Production of Biochar

#### 2.2.1. Pretreatment of Precursors

Before converting biomass into biochar, simple pretreatment methods can increase BC yield, optimize its characteristics, and remove impurities [44]. The primary pretreatment methods include physical pretreatment (e.g., ball milling, heat treatment) [45,46], chemical pretreatment (e.g., oxidation, acid and alkali solution soaking) [47,48,49,50], and biological pretreatment (e.g., enzymatic degradation, anaerobic digestion) [51,52]. For instance, in the study by Wang et al., a multistage gradient structure of N, S co-doped carbon was prepared by soaking the precursor in KOH, resulting in BC with abundant mesopores and a high specific surface area [47]. Yang et al. employed anaerobic digestion of biomass, which created favorable conditions for nitrogen incorporation. The BC produced from the anaerobically digested biomass exhibited superior physicochemical properties compared to BC derived from virgin biomass, including higher specific surface area, nitrogen content, and degree of graphitization [51].

#### 2.2.2. Activation of Biochar

BC activation can enhance its physical structure, introduce functional groups, and create conditions suitable for material loading [53]. Common activation methods include physical activation (e.g., ball milling, heat treatment) [7,54,55,56], chemical activation (e.g., acid activation, alkali activation, metal oxide activation) [57,58], and physicochemical co-activation [59,60]. For example, Xu et al. applied KOH and KMnO_4_ to co-activate wheat straw BC, which resulted in BC with a higher specific surface area and a richer pore structure [58].

#### 2.2.3. Synthesis Methods of Biochar

BC can be prepared using various methods, with pyrolysis (including fast pyrolysis and slow pyrolysis) being the most common. Other preparation methods include gasification, hydrothermal carbonization, microwave pyrolysis, flash carbonization, and baking [61]. However, the chars obtained from gasification and hydrothermal carbonization typically do not meet the definition of BC [62]. This section summarizes a few common preparation methods and the conditions under which they are carried out as shown in Table 1.

### 2.3. Structural Characteristics of Biochar

BC is produced from biomass via pyrolysis and carbonization, resulting in a carbon-rich microporous material with high aromaticity. Its structural properties depend on pyrolysis temperature, mode, and precursor characteristics [74]. Higher pyrolysis temperatures increase BC porosity, enhancing its adsorption capacity [75,76]. Ouyang et al. found that elevated temperatures can lead to the collapse of the carbon skeleton and increased activation capacity for peroxymonosulfate (PMS) [77]. However, repeated experiments may oxidize the framework and defect edges, increasing oxygen-containing functional groups, which reduces its electron transfer efficiency to PMS. While the aromatic structure and nanopore size generally increase with carbonization temperature, temperatures above 700 °C can damage microporous structures, and above 800 °C, the carbon skeleton may become unstable [78,79]. The structure of BC also varies based on the biomass source and cellulose and lignin content [80]. Abundant hydroxyl groups in cellulose and hemicellulose contribute to micropore formation, resulting in a three-dimensional structure [81]. This extensive pore structure and stability make lignocellulosic BC effective for metal loading and suitable for complex water treatment systems [26].

## 3. Application of Biochar in the Degradation of Wastewater Pollutants

### 3.1. Application of Biochar in Adsorption System

The adsorption process is a key method for wastewater treatment [82]. While activated carbon is widely used due to its large surface area and thermal stability [83], its high cost and regeneration difficulties have led to a search for more economical alternatives [84]. BC, derived from agricultural waste and municipal sludge, offers low cost, easy modification, and substantial microporosity, exemplifying “waste for waste” in pollutant adsorption [85]. Table 2 summarizes BC’s application for degrading pollutants in the adsorption system.

#### 3.1.1. Native Biochar Adsorbents

**Figure 2:**
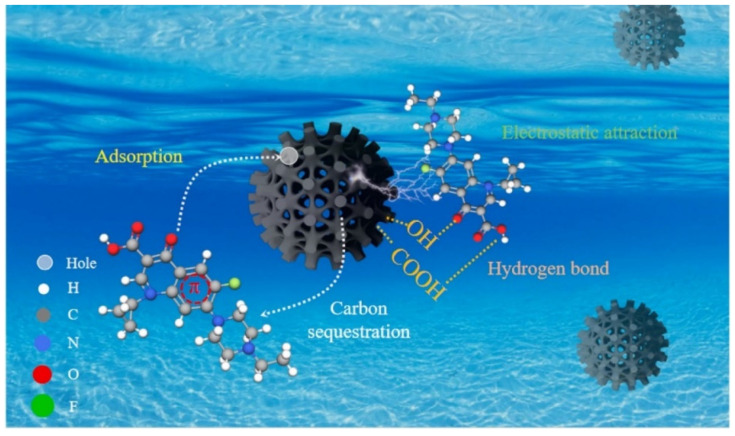
Mechanisms responsible for adsorption of enrofloxacin on BCs. (Reproduced from [86]).

BC is considered a sustainable substitute for activated carbon, effectively removing various pollutants from wastewater [96]. Researchers have explored different surface modification methods to enhance BC’s adsorption capacity. For instance, Jiang et al. activated BC derived from cow dung using varying concentrations of H_3_PO_4_, achieving an enrofloxacin adsorption capacity of 63.61 mg/g, six times higher than that of unmodified BC (12.66 mg/g) [86]. This phosphoric acid treatment enriched the BC’s pores and increased its specific surface area, significantly improving its adsorption performance. The modified BC exhibited numerous functional groups, such as hydroxyl, carboxyl, and carbonyl groups, which facilitated hydrogen bonding with enrofloxacin through π–π interactions, enhancing the hydrophobic effect (Figure 2). Alkali modification is another effective approach for boosting BC’s adsorption capabilities. Xu et al. combined KOH and KMnO_4_ to increase the maximum tetracycline (TC) adsorption capacity to 584.19 mg/g [58]. The KMnO_4_ modification promoted mesopore formation, while KOH enhanced micropore generation, significantly improving the physical and chemical properties of the BC. After modification, the I_D_/I_G_ ratio decreased from 2.58 to 2.32, indicating enhanced π–π interactions and charge transfer during TC adsorption.

Physical modifications, such as ball milling, are also popular due to their environmentally friendly nature. This method, known for its simplicity and efficiency, reduces solid materials to the nanometer scale, increasing specific surface area and creating new interfaces [97]. Zhang et al. tested ball-milled BC from various biomass sources and found that ball milling significantly improved the physicochemical properties and adsorption capacity of galaxolide [55]. Although the increase in oxygen-containing functional groups slightly decreased hydrophobicity, the overall gain in adsorption capacity outweighed this loss. Harindintwali et al. reported that ball milling enhanced BC’s adsorption efficiency for both organic and inorganic pollutants by increasing specific surface area, pore volume, and acidic oxygen functional groups, as well as improving negative zeta potential. This process enhances adsorption through surface complexation, electrostatic attraction, pore filling, and π–π interactions [98].

#### 3.1.2. Metal-Doped Biochar Adsorbents

**Figure 3:**
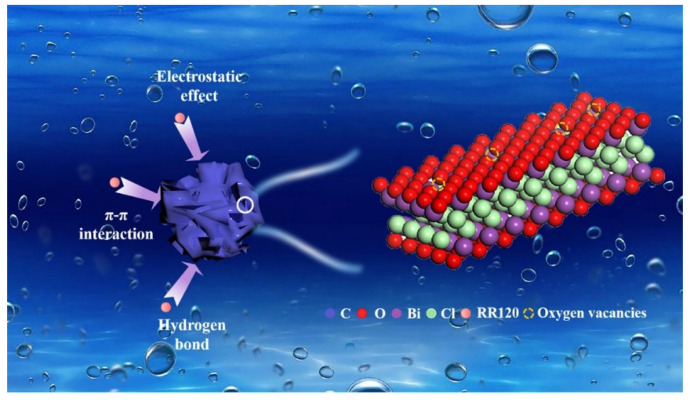
Schematic diagram of adsorption mechanism for RR120 on 50%-BiOCl/BC. (Reproduced from [89]).

Recently, metal compounds/BC composites have gained popularity in adsorption technology. The addition of metal dopants can significantly enhance the adsorption capacity of BC [99]. Zhuo et al. modified corn stover-derived BC with calcium chloride, resulting in a nearly twofold increase in its tetracycline adsorption capacity [87]. Similarly, Luo et al. created bismuth oxychloride/BC (BiOCl/BC) nanocomposites via ball milling, which exhibited excellent adsorption performance for both water and reactive red-120 (RR120), with a maximum capacity of 116.382 mg/g [89]. Characterization showed that BiOCl/BC was rich in oxygen vacancies, providing abundant adsorption sites. Electrostatic and π–π interactions also significantly contributed to the adsorption process (Figure 3). The high adsorption rate under acidic conditions, contrasted with a lower rate in alkaline conditions, highlighted the importance of electrostatic attraction between the sulfonic acid group of RR120 and the BiOCl/BC surface. Thus, the combination of high specific surface area, abundant oxygen vacancies, and multiple interactions accounts for the superior adsorption capacity of BiOCl/BC.

Magnetic adsorbents not only offer recyclability but also demonstrate improved pollutant removal compared to conventional BC. Metals such as cobalt, nickel, and iron can be incorporated into carbon-based materials through methods like microwave synthesis, co-precipitation, and hydrothermal processes [100,101]. For example, Zhang et al. developed magnetized BC (MF-CMS) with a spherical structure and a specific surface area of 747.76 m^2^/g, achieving a 96.02% removal rate for tetracycline [91]. Additionally, Li et al. created a novel surface-imprinted polymer (MIP-MBC) for targeted adsorption of sulfamethoxazole (SMX) using Fe-Mn modified BC, which reached a maximum adsorption capacity of 25.65 mg/g—1.34 times higher than that of the non-imprinted magnetic BC [92]. The mesoporous structure and oxygen-containing functional groups significantly enhanced the adsorption efficiency of SMX.

#### 3.1.3. Heteroatom-Doped Biochar Adsorbents

In addition to acid and alkali modifications, as well as metal oxide/BC composites [60], heteroatom doping significantly enhances BC’s adsorption capacity. Nitrogen doping improves graphitization, increases oxygen- and nitrogen-containing functional groups, and enhances hydrophilicity, boosting adsorption efficiency [102]. However, excessive nitrogen doping can reduce adsorption capacity by increasing surface nitrogen content and the I_D_/I_G_ ratio while decreasing surface oxygen content, which is critical for certain pollutants [95]. BC adsorbs pollutants via π–π interactions and weak electrostatic forces due to its rich surface functional groups. Nitrogen doping and defects enhance this capacity [16]. The ball milling method for nitrogen-doped BC is gaining attention for its simplicity and low energy use. Wu et al. created urea N-doped BC with an adsorption capacity of 11.48 mg/g for norfloxacin at pH ≈ 5, significantly higher than the 2.83 mg/g of unmodified BC [93]. This improvement was effective across a pH range of 3–9, driven by enhanced hydrogen bonding, π–π interactions, and pore filling. Ma et al. developed an optimally hydrothermal N-doped sludge BC using melamine, resulting in BNSBC-0.5 [7]. Analyses showed that BNSBC-0.5 adsorbs sulfamethoxazole (SMX) mainly through Lewis acid-base interactions, π–π conjugation, pore filling, and electrostatic interactions, achieving over 88% removal efficiency from various water sources.

### 3.2. Application of Biochar-Based Catalysts in Fenton-like Systems

The adsorption process transfers contaminants without eliminating them, leading to BC saturation that requires periodic replacement, thus increasing treatment costs. In contrast, Fenton-like systems generate free radicals that mineralize contaminants into harmless water and carbon dioxide, making them effective for complex pollutants resistant to adsorption [103]. BC’s abundant functional groups and vacancies activate oxidants to produce free radicals, while its pore structure supports metal loading and complex catalysis. Table 3 summarizes BC’s application for degrading pollutants in Fenton-like systems.

#### 3.2.1. Native Biochar-Based Catalysts in Fenton-like Systems

**Figure 4:**
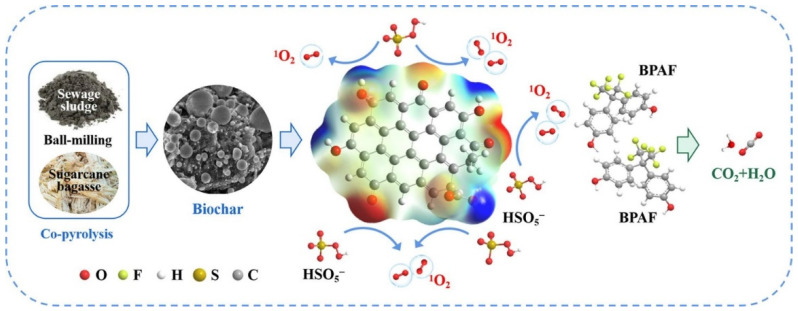
Schematic diagram of the mechanism of OFGs activating PMS to produce single linear oxygen to degrade pollutants. (Reproduced from [106]).

BC contains PFRs and OFGs that activate persulfate (PS) and H_2_O_2_ to generate HO^•^ [125]. Fang et al. showed that BC from pine needles, wheat, and corn stover activated H_2_O_2_ to degrade dichlorobiphenyl, with PFRs identified as key contributors to HO^•^ formation [126]. Li et al. created a novel BC catalyst, SB-BC-900, from co-pyrolyzed sewage sludge and bagasse, which effectively activated peroxomonosulfate (PMS) for bisphenol AF (BPAF) degradation [106]. XRD showed decreased graphitization of SB-BC-900 post-reaction, while FTIR and X-ray photoelectron spectroscopy (XPS) analyses indicated reduced C=O and C=C peaks, highlighting the influence of sp^2^ carbon and OFGs (Figure 4).

Structural defects also impact catalytic efficiency. Research shows that BC prepared at high temperatures exhibits varying catalytic capacities based on its defective structures (e.g., edge defects, curvature defects, and vacancies) [77,105,127]. For instance, Zhang et al. found that although environmentally persistent free radicals (EPFRs) diminished at elevated temperatures (800–900 °C), BC’s activation capacity remained high due to the presence of these defects [104]. XPS analysis of pine BC revealed changes in C-C, C-C/C-H, and C-O bond ratios before and after reactions, underscoring the importance of defective structures in reactions. The proportion of C-C bonds decreased from 71.75% to 67.40%, while C-C/C-H bonds increased from 11.73% to 19.89%. Research shows that higher preparation temperatures correlate with more defects and enhanced activation capacity, making the optimization of defect structures a key focus for improving BC’s catalytic performance [128].

#### 3.2.2. Metal-Based Biochar Catalysts in Fenton-like Systems

##### Monometallic-Based Biochar Catalysts

Iron’s catalytic, magnetic, and reducing properties make BC-Fe composites effective by combining BC with iron [129]. Nano-zero-valent iron (nZVI) has been widely studied as a substitute for Fe^2+^ in Fenton systems [130,131]. However, nZVI tends to aggregate into microparticles due to magnetic interactions and high surface energy, limiting its catalytic efficiency. BC can stabilize nZVI, enhancing its performance due to its porous structure. Zhu et al. combined nZVI with nickel to create composites that degrade pollutants like norfloxacin by promoting Fe ion cycling [132]. Metal aggregation can be effectively inhibited by ball-milling transition metals with BC. He et al. prepared FeS@BC by ball milling, which successfully inhibited the aggregation of FeS and facilitated the electron transfer between PS, the electron donor, and the pollutant, which led to the non-radical degradation of the pollutant [122].

Copper oxide (CuO) has also been widely studied as a transition metal oxide catalyst in the activation of PDS. Li et al. developed RSBC-CuO composites through hydrothermal methods, which degraded finasteride (PNT) over a wide pH range (4.26~10), mainly relying on Cu^3+^ activation and PDS-driven HO^•^ and SO_4_^•−^ radical generation [111]. Similarly, Zhao et al. created e-CuO@BC catalysts, which selectively degraded electron-rich pollutants via Cu-O-C bonding and enhanced PDS activation [133]. BC-CuO/PMS systems also proved effective in treating high-salt wastewater [134].

Cobalt (Co) is highly effective in activating PS [135,136,137], though cobalt leaching poses potential health risks. BC’s carbon-rich structure provides an ideal cobalt carrier, offering excellent electrical conductivity, stability, low cost, and environmental friendliness [138,139,140]. Song et al. developed a Co_3_O_4_-loaded BC (Bio-Co_3_O_4_) from peanut shells, which effectively catalyzed PMS-driven antibiotic degradation in wastewater. The BC prevented Co_3_O_4_ agglomeration, increased active sites, and improved stability over multiple uses [116]. Additionally, the metal introduction enhanced pore size and surface area, aiding metal loading and minimizing leaching risk [140,141]. The release of cobalt ions from BC-Co_3_O_4_ was below 0.1 mg·L^−1^, far below the WHO drinking water standard of 10 μg/L.

##### Multimetal-Based Biochar Catalysts

Bimetallic and polymetallic catalysts often perform better than monometallic ones [142]. Various bimetallic compounds are commonly used [26,77,143,144,145]. In spinel-based BC catalysts, doped metals not only provide catalytic activity but also create defective structures that enhance performance. Wang et al. discovered that oxygen defects and C=O groups on doped bimetallic oxides significantly boost their catalytic abilities [146]. Li et al. explored metal doping further, preparing a CoFe_2_O_4_-loaded BC composite (CFB-2) to degrade tetracycline hydrochloride (TCH) by activating peroxyacetic acid [121]. When K_2_Cr_2_O_7_, an electron scavenger, was added, the TCH removal was almost completely inhibited, suggesting that C=O groups act as Lewis bases, facilitating electron transfer and redox reactions that generate singlet oxygen (^1^O_2_). Cyclic voltammetry revealed larger current peaks on CFB-2 compared to BC alone, indicating improved electron transfer and ROS generation.

#### 3.2.3. Heteroatom-Doped Biochar Catalysts in Fenton-like Systems

**Figure 5:**
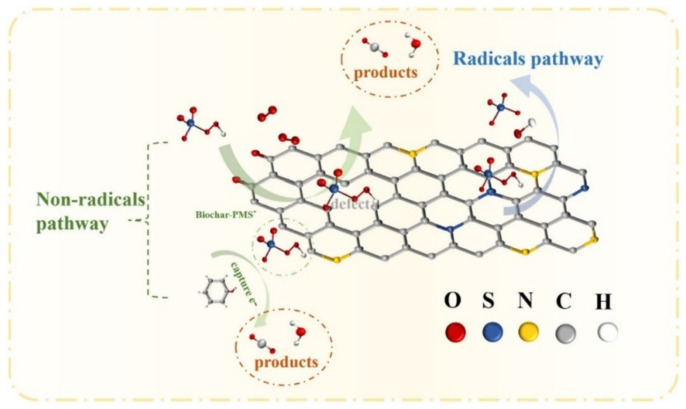
Proposed mechanism for the degradation of phenol. (Reprinted from [28]).

Metal leaching is a drawback of metal catalysts, so metal-free, carbon-based materials are being developed for PMS activation. Heteroatom doping (e.g., nitrogen, sulfur) has been shown to significantly improve PMS activation efficiency [147]. However, carbon materials are often inert, limiting their catalytic performance. Ye et al. found that nitrogen-doped, graphitized BC catalysts effectively degrade pollutants through a non-radical pathway, which is less affected by complex wastewater compositions [123]. Fan et al. used Ginkgo biloba leaves to prepare BC that efficiently activated PMS for phenol degradation [28]. The results showed that PMS adsorption was facilitated by oxygen, sulfur, and nitrogen groups on the BC. Sulfur functional groups increase the number of positively charged carbon atoms, promoting O-O bond cleavage and radical generation during PMS activation. Additionally, PMS forms adducts with C-O groups, which then produce ^1^O_2_ for the non-radical degradation of phenol (Figure 5).

### 3.3. Application of Biochar-Based Catalysts in Photocatalytic Systems

Under sunlight, the BC carbon matrix (BCM) and the dissolved organic matter (DOM) it releases promote the production of HO^•^ and ^1^O_2_ through light-induced energy and electron transfer [100]. BC also enhances light absorption [148], reduces electron-hole pair recombination [149], and increases catalyst surface area, making it an effective photocatalyst carrier. Recent advances in BC-based photocatalysis are significant, as summarized in Table 4.

#### 3.3.1. Native Biochar-Based Catalysts in Photocatalytic Systems

Fang et al. studied the photochemical activity of BC and the role of PFRs in HO^•^ production [162]. They found that DOM-rich BC enhanced ^1^O_2_ formation, while BC with abundant surface PFRs increased HO^•^ generation under light. By comparing BC from different sources (e.g., pine needles, and wheat straw), they concluded that PFRs contributed to over 60% of HO^•^ production, highlighting PFRs as key factors in photocatalytic systems. Wan et al. examined reactive species generated by straw BC-derived dissolved black carbon (DBC) during photocatalysis [163]. Through transient absorption spectra of rice DBC under 355 nm laser irradiation, an absorption band around 440 nm was attributed to the production of the DBC triplet state (^3^DBC*). The high quantum yield and electron transfer capacity of ^3^DBC* accelerated pollutant degradation in DBC-rich water. Electron spin resonance experiments confirmed the presence of both HO^•^ and ^1^O_2_.

#### 3.3.2. Semiconductor-Doped Biochar-Based Catalysts in Photocatalytic Systems

##### TiO_2_/Biochar-Based Catalysts

**Figure 6:**
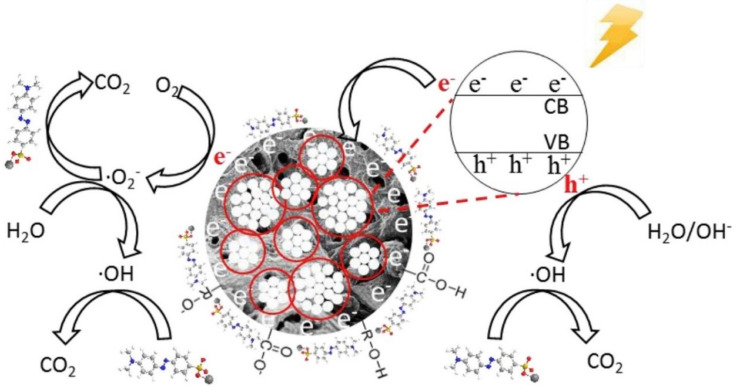
Schematic illustration of the catalytic degradation of MO. (Reprinted from [151]).

TiO_2_ is one of the first photocatalytic materials extensively studied [164]. However, its large band gap (E_g_ = 3.2 eV) limits excitation to UV or near-UV radiation [165], and the rapid recombination of photogenerated electrons and holes significantly reduces photocatalytic efficiency [166]. Carbonaceous nanomaterials can serve as electron carriers and transport channels, enhancing photocatalytic performance [167]. Lu et al. synthesized TiO_2_/BC composite catalysts by hydrolyzing TiO_2_ deposited on BC [151]. Under UV irradiation, the composite catalyst achieved a decolorization efficiency of 96.88%, outperforming the TiO_2_ catalyst alone (76.69%). Quenching experiments indicated that holes, HO^•^, and superoxide anions (O_2_^•−^) were involved in decolorization, with HO^•^ being the most significant contributor. Under UV light, TiO_2_ generates electrons and holes, while BC transfers electrons to minimize recombination. The electrons then react with oxygen molecules to produce O_2_^•−^, which generates HO^•^. Additionally, water reacts with holes to form HO^•^. Ultimately, these reactive species degrade methyl orange (MO) into CO_2_ and water (Figure 6). Recently, ball milling has been increasingly applied in photocatalysis. Mancuso et al. prepared TiO_2_/BC composites (C/TiO_2_)BM for tannery wastewater treatment using this method [168]. Characterization showed that ball milling created oxygen vacancies and enhanced the graphitization of BC, improving electron transfer conditions and contributing to superior dye degradation performance compared to TiO_2_ alone.

##### Graphite (g-C_3_N_4_)/Biochar-Based Catalysts

**Figure 7:**
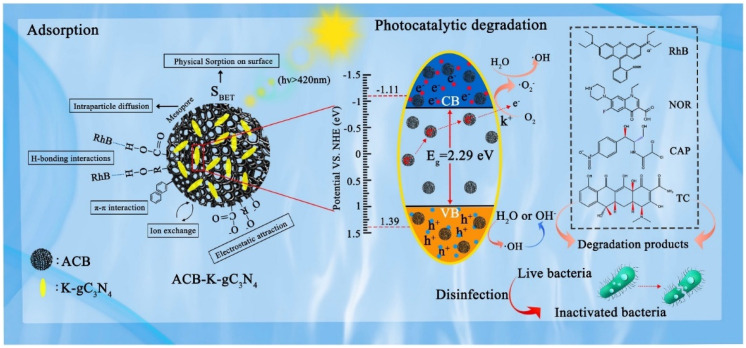
Schematic illustration of the possible adsorption-photocatalytic mechanisms of ACB-K-gC_3_N_4_ for pollutant degradation and disinfection under visible light irradiation. (Reprinted from [175]).

g-C_3_N_4_, with its conjugated structure and semiconductor properties, effectively absorbs visible light to generate photoelectron-activated oxidants, which promote ROS for water pollutant degradation [169,170,171,172]. However, it faces challenges such as high electron-hole recombination efficiency, low visible light utilization, and limited surface area [173]. In contrast, BC offers excellent electrical conductivity and electron storage, enhancing g-C_3_N_4_’s photocatalytic performance by reducing electron-hole recombination. Sun et al. created a BC-modified hybrid photocatalyst with g-C_3_N_4_ and ferrite using bamboo fibers [174]. This catalyst achieved over 96.7% degradation of MO in 2 h under visible light with hydrogen peroxide. Compared to pure g-C_3_N_4_, it demonstrated a smaller band gap, lower electron transport resistance, and improved visible light absorption. Wang et al. used ultrasonic milling and KOH to activate BC, preparing ACB-K-gC_3_N_4_ [175]. Its photodegradation mechanism involved the oxidation of O_2_^•−^, holes, and HO^•^, enhanced by the activated BC’s properties. Additionally, ACB and K^+^ ions improved electron transfer while preventing carrier recombination (Figure 7). Intimately coupled photocatalysis and biodegradation (ICPB) effectively remove persistent pollutants. Zhang et al. developed BC/g-C_3_N_4_ as a porous hydrogel for the ICPB system, achieving a tetracycline hydrochloride (TCH) removal rate of 96.0%, outperforming other systems [176]. Microorganisms facilitated pollutant degradation and enhanced TCH mineralization.

#### 3.3.3. Heteroatom-Doped Biochar Catalysts in Photocatalytic Systems

Literature studies have shown that doping of BC with heteroatoms (S, C, N, etc.) enhances photosensitivity, increases photogenerated electron-hole pairs, and narrows semiconductor bandwidth [159,177]. Xiong et al. prepared nitrogen-doped TiO_2_ BC catalysts for the photodegradation of methylene blue, achieving a removal rate of 97.6% compared to 52.6% for undoped TiO_2_ [152]. XPS revealed nitrogen as substituted organic phases, creating new impurity energy levels that reduced the energy band gap and improved photocatalytic activity. Nitrogen incorporated into the TiO_2_ lattice decreased the energy band gap and enhanced visible light responsiveness. Sun et al. noted that nitrogen vacancies in BC/g-C_3_N_4_ composite catalysts, combined with bridging C and N vacancies, facilitated the separation and migration of photogenerated carriers [178]. Wang et al. utilized iodine doping to create visible light-induced BC photocatalysts, resulting in first-order degradation kinetics that were 24.7 and 23.7 times faster than undoped BC [179]. Iodine doping generated new micropores and mesopores, enhancing pollutant adsorption and subsequent degradation. It also narrowed the band gap and reduced electron transfer resistance, improving photocatalytic activity.

#### 3.3.4. Composite Biochar-Based Catalysts in Photocatalytic Systems

The integration of BC with metallic photocatalysts has been widely explored. Yang et al. synthesized BiOBr/lignin-BC composites with oxygen vacancies to degrade rhodamine B via hydrothermal methods [160]. C doping increased oxygen vacancies and improved BiOBr’s visible light absorption, reducing carrier recombination. UV-visible diffuse reflectance spectroscopy showed that lignin enhanced BiOBr’s light absorption and facilitated electron-hole separation. Strong interactions between BiOBr and lignin-BC reduced charge transport resistance, improving separation efficiency and accelerating degradation. The electron-absorbing properties of BC enabled rapid electron transfer to surface-adsorbed O_2_, forming O_2_^•−^. Hosny et al. developed silver/ZnO@BC nanocomposites for tetracycline degradation using sonication and pyrolysis [180]. Under optimal conditions, silver/ZnO@BC achieved a degradation efficiency of 70.3%. UV irradiation generated electron-hole pairs, with free electrons reacting with oxygen to form ROS like O_2_^•−^ and HO^•^. Additionally, Ag/ZnO@BC showed effectiveness as an antimicrobial agent against Klebsiella pneumoniae and as a potential antioxidant material.

### 3.4. Application of Biochar-Based Catalysts in Sonocatalytic Systems

Ultrasonic irradiation induces localized pressure changes in liquids, causing bubble expansion, contraction, or rupture, which results in transient cavitation. This generates high temperatures (up to 3000 K) and pressures that produce HO^•^ from water molecules [181]. However, this acoustochemical process is often inefficient, with long processing times and high energy consumption. Studies show that carbon particles in the liquid phase can significantly enhance pollutant adsorption and degradation through ultrasound-induced surface activation [182,183,184,185]. Thus, BC-based catalysts can address these limitations [125]. Table 5 summarizes their applications in acoustic catalytic systems.

#### 3.4.1. Native Biochar-Based Catalysts in Sonocatalytic Systems

Nikolaou et al. assessed the impact of rice husk BC on propyl hydroxybenzoate (PP) degradation under 20 kHz ultrasonic irradiation [183]. Results showed that PP removal was only 15% without BC but increased to about 70% with 125 mg·L^−1^ BC. Kim et al. found that at 300 kHz, BC exhibited low dispersion; therefore, the ultrasonic effect was stronger at 40 kHz, yielding a higher rhodamine B removal rate of 51.8% compared to 26.2% at 300 kHz [182]. ROS generation from the BC-based catalyst is detailed in Equations. (1)–(9). Li et al. introduced cetyltrimethylammonium bromide (CTAB) to study its effects on the ultrasonic degradation of Orange II (OR II) by bagasse BC [190]. Results indicated that high CTAB concentrations or increased bagasse BC significantly enhanced catalytic efficiency. The synergistic effect of CTAB and BC improved degradation performance, with quenching experiments confirming the dominant role of HO^•^. The degradation mechanism of OR II on the BC surface involves three steps: first, CTAB forms a complex with BC; second, negatively charged OR II is adsorbed onto the positively charged BC; and third, HO^•^ generated on the surface degrades OR II, with enhanced adsorption accelerating this reaction.

(1) $$H_{2}O+ulstrasound\to HO^{•}+H^{•}$$

(2) $$BC{\left(\mathrm{metals}\right)}^{+}\to BC\left(\mathrm{metals}\right)-e^{-}$$

(3) $$BC\left(\mathrm{metals}\right)-e^{-}+O_{2}\to BC\left(\mathrm{metals}\right)+{O_{2}}^{•-}$$

(4) $${O_{2}}^{•-}+H^{+}\to H{O_{2}}^{•-}$$

(5) $$H{O_{2}}^{•}+{O_{2}}^{•-}+H^{+}\to H_{2}O_{2}+O_{2}$$

(6) $$H_{2}O_{2}+{e_{CB}}^{-}\to HO^{•}+OH^{-}$$

(7) $$H{O_{2}}^{•}+{O_{2}}^{•-}\to HO^{•}+OH^{-}+O_{2}$$

(8) $$H_{2}O+{h_{VB}}^{+}\to OH^{•}+H^{+}$$

(9) $$OH^{-}+{h_{VB}}^{+}\to OH^{•}$$

#### 3.4.2. Composite-Based Biochar Catalysts in Sonocatalytic Systems

Cerium oxide (CeO_2_), a lanthanide metal oxide, can undergo redox cycling between +4 and +3 valences, making it a versatile catalyst for degrading organic pollutants [191]. BC-CeO_2_ nanoparticles, synthesized via hydrothermal methods by Khataee et al., demonstrated excellent acoustic catalytic performance, improving pollutant mass transfer from liquid to catalyst surface, leading to higher removal rates than single CeO_2_-H or BC alone [192]. The acoustic catalysis process generated a broad wavelength range of UV light, exciting CeO_2_ to produce HO^•^, while BC facilitated electron transfer at the CeO_2_ interface, preventing electron-hole recombination.

**Figure 8:**
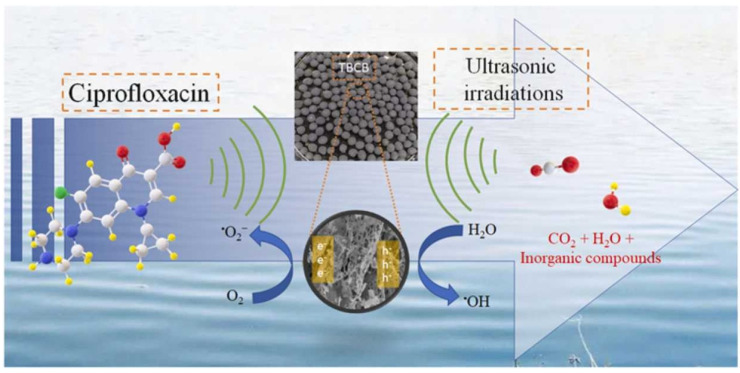
Possible mechanism of CIP degradation by TBCB in the sonocatalytic systems. (Reprinted from [186]).

Semiconductors, particularly when combined with BC, significantly enhance pollutant removal in acoustic catalysis [193]. Afzal et al. developed TiO_2_-encapsulated BC beads using pyrolyzed agricultural waste and chitosan for the effective degradation of ciprofloxacin (CIP) [186]. CIP degradation depended on ROS generated through acoustic catalysis, with HO^•^ being predominant. Microbubble implosion during sonication created high pressures (~1800 atm) and temperatures (up to 5000 K), dissociating water molecules to generate HO^•^. This process allowed TiO_2_ electrons to mobilize efficiently, enhancing the production of HO^•^ and O_2_^•−^, which further degraded CIP (Figure 8). Gholami et al. synthesized Fe-Cu layered double hydroxide/BC nanocomposites to study their ultrasonic degradation activity for cefazolin sodium (CFZ), achieving a 97.6% degradation rate within 80 min after optimization [188]. Quenching experiments confirmed the dominant role of HO^•^ in CFZ degradation.

### 3.5. Application of Biochar-Based Catalysts in Electrocatalytic Systems

Electrochemical advanced oxidation processes (EAOPs) rely on oxidation reactions at the anode, generating oxygen through water electrolysis. Electrons are transferred to the cathode to produce H_2_O_2_ via a two-electron oxygen reduction reaction (2e−ORR), which is subsequently catalyzed to generate HO^•^ radicals capable of degrading organic pollutants [194]. Electrode material selection critically affects current efficiency, H_2_O_2_ generation, selectivity, mechanism, and pollutant degradation efficiency [195]. Anode materials should possess catalytic activity and stability, including NiMnO_3_, Fe_2_O_3_, Ti/metal oxides, and carbon/graphite felts [196,197,198,199]. Various cathode materials have been developed to enhance in situ H_2_O_2_ generation, including graphite felts, noble metals (e.g., Pt, Au, Pd), and metal oxides (e.g., CeO_2_, SnO_2_) [200,201,202,203]. However, these materials can be costly and unsustainable. BC’s physicochemical properties, such as high conductivity and large surface area, enhance its use in EAOPs [204]. Table 6 summarizes BC applications in electrocatalysis.

#### 3.5.1. Native Biochar Electrode in Electrocatalytic Systems

Ansari et al. evaluated a stainless steel mesh-wrapped banana-peel-derived BC (BB) cathode for in situ H_2_O_2_ generation, targeting the degradation of bromophenol blue (BPB) and Congo red (CR) dyes [208]. Up to 9.4 mg·L^−1^ of H_2_O_2_ was generated after 60 min using 2.0 g of BB at a current of 100 mA under pH-neutral conditions, achieving removal efficiencies of 87.44% for BPB and 83.63% for CR. Kim et al. employed banana-peel-derived BC (BP-BC) in a continuous-flow reactor for ibuprofen (IBP) degradation [209]. The BP-BC cathode produced H_2_O_2_ via 2e−ORR, catalyzing its decomposition to generate HO^•^, which oxidized IBP. However, limited surface functional groups restricted H_2_O_2_ generation (~3.4 mg·mL^−1^), resulting in only ~40% IBP degradation. The addition of PS significantly improved IBP removal, enabling the co-generation of HO^•^ and SO_4_^•−^ radicals, achieving nearly 100% degradation. Ma et al. incorporated ball-milled, acetic acid-modified sludge BC (BASBC) into wastewater, establishing an electrocatalytic system that resulted in 86.1% mineralization efficiency for imidacloprid [206]. BASBC provided abundant catalytic sites, activating PS directly while supplying electrons for continuous generation of -COOH and -OH groups, enhancing PS activation to produce more ROS.

#### 3.5.2. Metal-Based Biochar Catalysts in Electrocatalytic Systems

Iron-carbon (Fe-C) microelectrolysis is an effective water treatment technology [216]. In this system, cast iron and activated carbon serve as electrode materials, where Fe^0^ loses electrons at the anode to generate Fe^2+^, while C accelerates reduction at the cathode [217]. Recent studies indicate that BC can replace activated carbon in Fe-C microelectrolysis systems. Li et al. developed catalytic Fe-C microelectrolysis granules (FeBCG) from Fe powder and sawdust, using them as PS activators to remove bisphenol A (BPA); optimal conditions yielded 100% removal within 20 min [218]. Burst, EPR, and electrochemical experiments demonstrated the involvement of SO_4_^•−^, O_2_^•−^, HO^•^, ^1^O_2_, and electron transfer in BPA removal, with chrono-current measurements indicating significant current changes upon PS and BPA addition, suggesting electron transfer between the FeBCG surface and both substrates. BC’s application in ferrocarbon microelectrolysis presents unique advantages, offering a cost-effective and efficient approach for developing BC-based electrocatalytic systems. Yu et al. utilized bimetallic-modified BC (MBC) as a cathode for tetracycline (TC) removal via PS activation, noting that the electric field enhanced the cathode’s catalytic activation by a factor of 2 to 5 [219]. The electric field boosted PS activation at specific sites on the catalytic surface, improving Mn^+^ regeneration efficiency.

#### 3.5.3. Heteroatom-Doped Biochar Catalysts in Electrocatalytic Systems

Nitrogen atoms, being more electronegative than carbon, induce positive charges on adjacent carbon atoms, facilitating O_2_ adsorption and reducing the overpotential of the oxygen reduction reaction [220]. Research shows that O-O bonding in perovskites involves O_2_ first adsorbing on positively charged carbon, then receiving electrons from nitrogen dopants, which lowers the energy threshold for O_2_ dissociation [221]. In electrocatalysis, nitrogen doping enhances the H_2_O_2_ yield of carbon-based cathodes [222]. Zhang et al. prepared nitrogen-doped BC from nitrogen-enriched coffee residues, creating a homogeneous air cathode (BCAC) for tetracycline (TC) degradation [214]. Experiments showed that BCAC-900 achieved 70.42% TC removal in 120 min at 4 mA cm⁻^2^. Free radical quenching experiments indicated that HO^•^ (37.36%), O_2_^•−^ (29.67%), and ^1^O_2_ (24.17%) were crucial for TC removal, with graphitic N acting as a key site for H_2_O_2_ generation and HO^•^ activation. Both graphitic N and nitrogen vacancies served as active sites for O_2_^•−^ and ^1^O_2_ generation. Luo et al. synthesized porous BC electrocatalysts co-doped with nitrogen and oxygen from peanut shells, achieving up to 92% H_2_O_2_ selectivity and an activation rate of 82% [223].

#### 3.5.4. Metal/Heteroatom-Doped Biochar Catalysts in Electrocatalytic Systems

**Figure 9:**
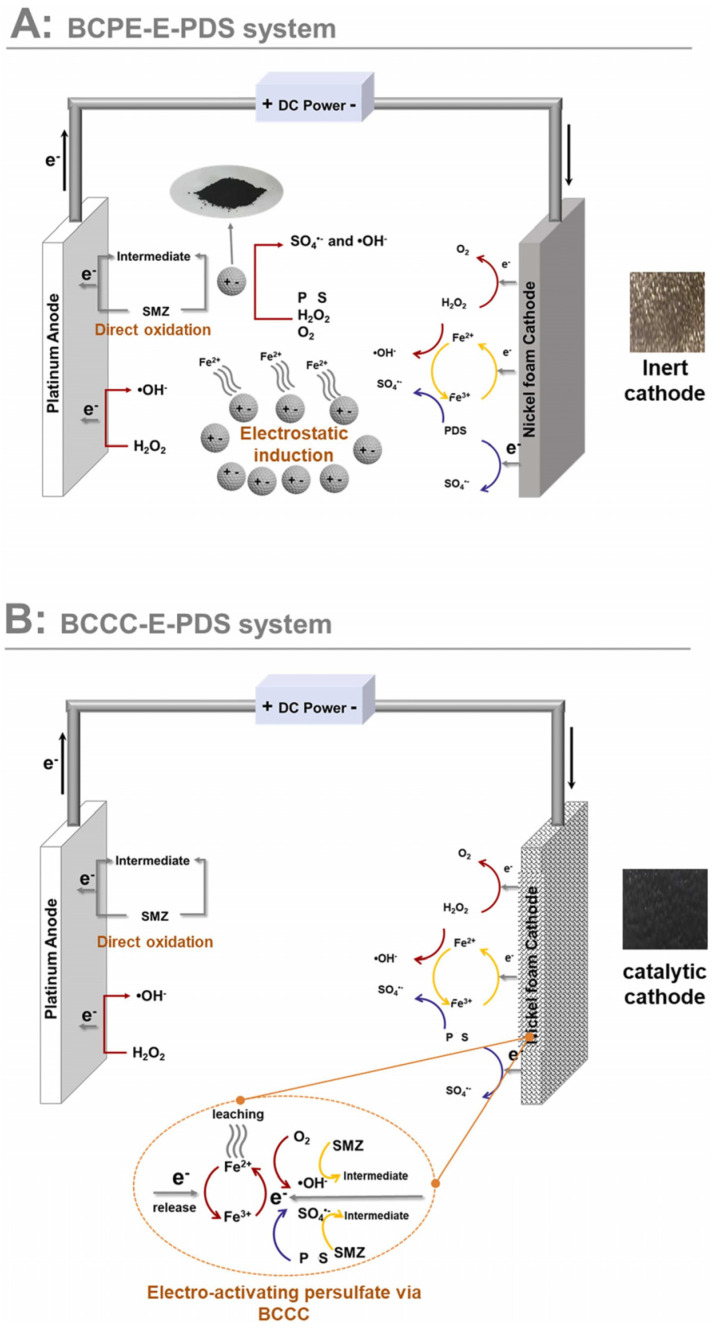
The proposed catalytic mechanism of BCCC and BCPE. (Reprinted from [210]).

Metal/heteroatom doping enhances the electrical conductivity and active sites of BC compared to undoped variants [204]. Ge et al. created a BC particle electrode (BCPE) from residual sludge and modified it on a nickel foam electrode to form a BC-catalyzed cathode (BCCC) for activating peroxodisulfate (PS) degradation from sulfonamide dimethylpyrimidine (SMZ) (Figure 9) [210]. They examined the catalytic performance of three-dimensional electrochemical systems (3DERs) with BCPE versus two-dimensional systems with BCCC. BC from pyrolyzed residual sludge was milled to create granular electrodes for the electrically activated PS reaction, involving PS (solution), nickel foam (cathode), and platinum (anode). Both BCPE and BCCC exhibited good catalytic performance, but their mechanisms differed. In the BCPE system, BCPE activated PS through electrostatic induction, while a small amount of Fe^2^⁺ also contributed. The BCCC system allowed more direct electron and energy transfer, enhancing SMZ oxidation. Zhang et al. developed Ti, Sn, and Ce-doped bamboo charcoal for 3DERs to efficiently treat coking wastewater [212]. The doping improved the electrooxidation and electrocatalytic properties, particularly with Ce enhancing HO^•^ generation. Additionally, BC can be made into electrocatalytic membranes. Yin et al. constructed Fe/N co-doped BC-based ultrafiltration membranes for the electrically assisted degradation of tetracycline and membrane fouling control [215]. The Fe-g-C_3_N_4_/ABC-600/Graphite/PVDF membrane achieved 100% tetracycline removal in 0.749 min and demonstrated excellent self-cleaning performance (100% flux recovery) with low energy consumption (0.199 W-h/L) in municipal wastewater treatment. Oxygen-rich functional groups and nitrogen species (e.g., pyridine N and pyrrole N) enhance H_2_O_2_ generation and accelerate Fe (III)/Fe (II) cycling, offering innovative pathways for developing advanced electrocatalytic systems.

## 4. Biochar Regeneration

BC can be regenerated after water treatment through chemical, biological, and physical methods. Chemical regeneration involves treating BC with acids, bases, or oxidizing agents (e.g., NaOH, HCl, H_2_O_2_) to remove adsorbed contaminants. This method is recognized as a sustainable technology that reduces both cost and energy consumption. The effectiveness of chemical regeneration depends on factors such as the adsorption mechanism, functional groups, the adsorbent’s pore structure, and changes in active adsorption sites [224]. Chemical regeneration is particularly effective for removing a wide range of pollutants, especially organic compounds and heavy metals [225,226,227,228]. However, high desorption efficiencies do not always correlate with high regeneration performance [229], and secondary toxic substances may adversely affect the stability of pollutants removed from the BC [224].

Biological regeneration is the process by which microorganisms renew the activated adsorbent sites in the BC’s pore structure [230]. Microorganisms utilize organic substrates from their metabolism to restore the adsorption capacity of the BC. This method is safer, more cost-effective, and environmentally friendly compared to steam and chemical regeneration [231]. However, bio-regeneration is influenced by factors such as dissolved oxygen levels and the structural characteristics of the BC [232].

Physical regeneration involves removing adsorbed contaminants from BC through thermal treatments (e.g., incineration or charring) or other physical methods such as steam vaporization [233,234,235]. During steam regeneration, steam rapidly heats the adsorbent bed, facilitating faster desorption of contaminants. However, desorption is only effective if the bed reaches a sufficiently high temperature to maintain the desorbate in the gaseous phase, allowing for efficient removal [231]. High-temperature regeneration involves complex physical and chemical processes influenced by numerous parameters. These factors directly impact the efficiency of carbon regeneration, which in turn determines the ability of BC to recover its adsorption capacity [236]. Additionally, thermally regenerated BC can be utilized for advanced oxidation processes, among other applications [237,238].

## 5. Pilot-Scale Biochar Water Treatment

Pilot-scale experiments are essential for evaluating the feasibility of applying biochar in practical settings. However, research on the removal of organic and inorganic pollutants in pilot-scale BC reactors is limited, with most studies focusing primarily on BC’s use as an adsorbent. James et al. investigated the impact of scaling up from laboratory- to pilot-scale production on BC quality, using a series of 100 mm × 1200 mm cylindrical treatment cells installed at the discharge point of an abandoned mine to facilitate pilot-scale BC deployment [239]. Under laboratory conditions, most BCs produced in the trial demonstrated higher zinc removal; however, when tested in the field, all BC exhibited a reduction in performance, ranging from 14% to 85%, depending on the BC type. Lnyang and Dickenson conducted a pilot study using three parallel adsorbents—granular activated carbon, anthracite, and hardwood BC—to treat tertiary filtration wastewater [240]. Granular activated carbon achieved the best reduction of perfluoropentanoic acid, perfluorohexanoic acid, perfluorooctanoic acid, perfluorooctanesulfonic acid, and dissolved organic carbon, followed by hardwood BC, with anthracite demonstrating the least effectiveness. These studies highlight the discrepancy between BC performance in laboratory-scale experiments and practical applications, underscoring the need for pilot-scale testing.

## 6. Conclusion and Prospects

The concept of using BC to treat wastewater has garnered significant interest among researchers. Employing low-cost and readily available BC as an adsorbent and catalyst for pollutant removal in various treatment systems marks an important advancement in water treatment. BC’s large surface area and high porosity provide excellent adsorption properties, while its PFRs and OFGs promote the generation of ROS and sulfate radicals. Its porous structure also allows for modifications (e.g., doping with transition metals and heteroatoms), enhancing its catalytic performance. As a result, BC has been effectively utilized in adsorption, Fenton-like reactions, photocatalysis, acoustic catalysis, and electrocatalysis for removing diverse pollutants. However, further research is needed to transition from experimental studies to large-scale applications.

Testing BC-based materials in real wastewater treatment is essential for their broader industrial adoption. Real wastewater is more complex than lab-simulated versions, containing a variety of pollutants and potential interferences. Research must focus on the stability and long-term reuse of BC under real wastewater conditions to ensure effectiveness and economic viability. Additionally, exploring the selective removal capabilities of BC for different contaminants is crucial for developing multifunctional wastewater treatment technologies.

The cost of implementing BC-based materials is a key factor for their large-scale promotion. These materials often need to be combined with other treatment technologies—such as electrocatalysis and photocatalysis—to improve efficiency, which can increase energy consumption and reagent costs. Therefore, developing BC-based systems should prioritize cost reduction and resource optimization to ensure feasibility in industrial applications. Moreover, challenges related to the recyclability of BC must be addressed. Low-energy and efficient catalytic systems will enhance the market competitiveness of BC and promote its use in wastewater treatment.

Machine learning (ML), a branch of artificial intelligence, can significantly reduce the workload and costs associated with trial-and-error in wastewater remediation [241,242]. For instance, ML models can be used to optimize the pyrolysis process and predict BC yield [243]. Additionally, ML can accurately forecast the pollutant removal efficiency of BC-based materials and identify optimal conditions for pollutant treatment [244]. Overall, machine learning holds considerable potential for advancing the application of biomass-derived BC in sustainable practices and the circular economy.

Despite their promising applications, the environmental and health impacts of BC-based materials must be considered. Studies indicate that BC can contain polycyclic aromatic hydrocarbons (PAHs) and trace heavy metals that may be released during treatment, potentially affecting human health and ecosystems. Future research should investigate the toxic effects of these substances and develop methods to minimize by-product generation, ensuring the safety and environmental friendliness of BC in practical wastewater treatment.

In conclusion, future research on BC-based materials for wastewater treatment should focus on the following directions: (1) increasing pilot-scale and industrial-scale studies to develop practical, recyclable, low-energy, and efficient pollutant removal systems; (2) investigating the toxicity of BC-based materials in real wastewater treatment applications and exploring conditions for toxicity regulation; and (3) employing ML to develop predictive models that optimize both the production conditions of BC-based materials and the wastewater treatment processes.

## Figures and Tables

**Table 1:** Common synthesis methods for biochar.

Synthesis Methods		Preparation Condition	Ref.
Pyrolysis	fast pyrolysis	400–800 °C, seconds	[63,64]
	slow pyrolysis	300–900 °C, minutes to days	[65,66]
Hydrothermal carbonization		180–250 °C, 0.5–48 h	[67,68,69,70]
Gasification		700–900 °C, 10–20 s	[71,72,73]

**Table 2:** Applications of BC-based adsorbents in the degradation of wastewater pollutants.

BC-Based Adsorbents	Targeted Pollutants	Operational Parameters	Q_max_ (mg/g)	Dominant Mechanism	Ref.
Phosphoric acid/cow dung BC	Enrofloxacin	IC^a^ = 20 mg/L; dosage = 0.32 g/L; pH = 5; t = 6 h, T = 25 °C	63.61	π–π interactions, pore filling, electrostatic interactions, and hydrogen bonding	[86]
KMnO_4_+KOH modified BC	Tetracycline	IC^a^ = 200 mg/L; dosage = 0.25 g/L; t = 24 h; T = 318 K	584.19	Pore filling, π–π interactions; hydrogen bonding, and metal complexation	[58]
Calcium modified BC	Tetracycline	IC^a^ = 20 mg/L; dosage = 1.0 g/L; pH = 8.0; t = 24 h	33.53	π–π interactions, and hydrogen bonding	[87]
ZnO/magnetic camphor leaves BC	Ciprofloxacin	IC^a^ = 100 mg/L; dosage = 0.5 g/L; pH = 4; t = 120 h; T = 40 °C	449.4	π–π interactions, electrostatic interactions, and cation exchange interactions	[88]
Iron oxide/banana-peel BC	Methylene blue	IC^a^ = 500 mg/L; dosage = 0.5 g/L; pH = 6.1; t = 12 h; T = 40 °C	862	An electron transfer process driven by electrostatic attraction	[19]
BiOCl/BC	Reactive red-120	IC^a^ = 10 mg/L; dosage = 0.4 g/L; pH = 2, t = 18 h; T = 25 °C	116.38	Electrostatic interactions, π–π interactions, and hydrogen bonding	[89]
ZnCl_2_/sunflower seed husk BC	Tetracycline	IC^a^ = 20 mg/L; dosage = 0.025 g/L; t = 24 h; T = 25 °C	673	Chemical and electrostatic interactions	[90]
Magnetic BC microsphere	Tetracycline	IC^a^ = 100 mg/L; dosage = 0.5 g/L; pH = 5; t = 24 h; T = 25 °C	94.63	Pore filling, electrostatic attraction, and π–π interactions	[91]
Surface-imprinted magnetic BC	Sulfamethoxazole	IC^a^ = 1000 mg/L; dosage = 1.0 g/L; t = 4 h; T = 35 °C	25.65	Hydrogen bonding, electrostatic interactions, and π–π interactions	[92]
Urea N-doped BC	Norfloxacin	IC^a^ = 50 mg/L; dosage = 1.0 g/L; pH = 5.0; t = 48 h	11.48	Hydrogen bonding, π–π electron donor-acceptor, and pore-filling interactions	[93]
N-doped/porous BC	Ciprofloxacin	IC^a^ = 30 mg/L; dosage = 0.125 g/L; pH = 6.58; t = 1 h; T = 30 °C	212	Combined filling pore, π–π conjugation, and hydrogen bonding.	[94]
N-doped/poplar BC	Phenol	IC^a^ = 100 mg/L; dosage = 0.2 g/L; t = 48 h; T = 25 °C	126.5–170.7	π–π stacking interactions	[95]
Hydrothermal N-doped sludge-derived BC	Sulfamethoxazole	IC^a^ = 15 mg/L; dosage = 0.0075 g/L; t = 12 h; T = 308 K	68.6	Lewis acid-base interactions, π–π conjugation, pore filling, and electrostatic interactions	[7]

IC^a^—initial concentration.

**Table 3:** Applications of BC-based catalysts in Fenton-like processes for the remediation of pollutants.

BC-Based Catalytic Systems	Targeted Pollutants	Operational Parameters	Reduction Efficiency	Dominant Radicals	Ref.
Pine BC+PMS	Tetracycline	IC^a^ = 20 mg/L; PMS dose = 3 mM; catalyst dosage = 3.0 g/L; pH = 7.0	90%	SO_4_^•−^ and HO^•^	[104]
Pine needle BC+PMS	1,4-dioxane	IC^a^ = 20 μM; PMS dose = 8 mM; catalyst dosage = 1 g/L; pH = 6.5	84.2%	SO_4_^•−^ and HO^•^	[77]
Corn cob BC+PDS	2,4-dichlorophenol	IC^a^ = 100 mg/L; PDS dose = 1 g/L; catalyst dosage = 0.2 g/L; pH = 6.0	86%	^1^O_2_	[105]
Soybean residue BC+PDS	Tetracycline hydrochloride	IC^a^ = 50 mg/L; PDS dose = 1 mM; catalyst dosage = 0.2 g/L; pH = 7	84.1%	^1^O_2_	[59]
Sludge-sugarcane bagasse BC+PMS	Bisphenol AF	IC^a^ = 20 mg/L; PMS dose = 50 mg/L; catalyst dosage = 0.2 g/L; pH = 7	93.7%	^1^O_2_	[106]
Red mud BC+PMS	Sulfamethoxazole	IC^a^ = 0.02 mM; PMS dose = 0.15 mM; catalyst dosage = 1.5 g/L; pH = 4.12	100%	^1^O_2_	[107]
Passion fruit shell BC+PMS	Tetracycline	IC^a^ = 20 mg/L; PMS dose = 0.3 g/L; catalyst dosage = 0.4 g/L; pH = 5.4	90.9%	^1^O_2_	[108]
Pine need BC+PMS	Phenol	IC^a^ = 10 mg/L; PMS dose = 3.0 mM; catalyst dosage = 0.2 g/L; pH = 5.2	100%	^1^O_2_, HO^•^ and SO_4_^•−^	[109]
Magnetic rape straw BC+PS	Tetracycline hydrochloride	IC^a^ = 20 mg/L; PMS dose = 8 mM; catalyst dosage = 1 g/L; pH = 5.68	98%	SO_4_^•−^ and ^1^O_2_	[110]
CuO/Rice straw BC+PDS	Phenacetin	IC^a^ = 10 mg/L; PDS dose = 50 mg/L; catalyst dosage = 0.3 g/L; pH = 4.26	100%	^1^O_2_ and O_2_^•−^	[111]
nano Fe_3_O_4_/Leaves BC+PMS	Bisphenol a	IC^a^ = 20 mg/L; PMS dose = 5 mM; catalyst dosage = 2 g/L; pH = 3	100%	SO_4_^•−^	[112]
Fe_3_O_4_/Porous BC+PMS	P-hydroxybenzoic acid	IC^a^ = 10 mg/L; PMS dose = 1 g/L; catalyst dosage = 0.2 g/L; pH = 8.5	100%	SO_4_^•−^	[113]
Co-doped/Shrimp shell BC+PMS	Ciprofloxacin	IC^a^ = 30 mg/L; PMS dose = 0.4 g/L; catalyst dosage = 0.15 g/L; pH = 6.8	89.5%	SO_4_^•−^ and O_2_^•−^	[114]
Co-doped/Goat manure BC+PMS	Ciprofloxacin	IC^a^ = 20 mg/L; PMS dose = 0.4 g/L; catalyst dosage = 0.1 g/L; pH = 6.3	96.5%	SO_4_^•−^, HO^•^ and O_2_^•−^	[115]
Co_3_O_4_/Peanut shell BC+PMS	Ofloxacin	IC^a^ = 20 mg/L; PMS dose = 1 mM; catalyst dosage = 0.8 g/L; pH = 7	97.3%	^1^O_2_ and SO_4_^•−^	[116]
Ni-doped/Cherry core BC+PMS	Bisphenol-A	IC^a^ = 20 mg/L; PMS dose = 1 g/L; catalyst dosage = 0.03 g/L; pH = 3	100%	SO_4_^•−^, HO^•^ and ^1^O_2_	[117]
Fe/Mn-doped/Sludge BC+PMS	Phenol	IC^a^ = 0.32 mM; PMS dose = 4 mM; catalyst dosage = 0.5 g/L; pH = 9	100%	^1^O_2_	[18]
MnFe_2_O_4_/BC+PMS	Bisphenol-A	IC^a^ = 20 mg/L; PMS dose = 0.2 g/L; catalyst dosage = 0.2 g/L; pH = 7.0	100%	O_2_^•−^ and ^1^O_2_	[118]
MgFe_2_O_4_/MgO/BC+PMS	Sulfamethoxazole	IC^a^ = 20 mg/L; PMS dose = 1 mM; catalyst dosage = 0.4 g/L; pH = 5.6	100%	^1^O_2_	[119]
CoWO_4_/Co-doped/BC+PMS	Chlortetracycline	IC^a^ = 20 mg/L; PMS dose = 0.3 mM; catalyst dosage = 0.03 g/L; pH = 5.2	100%	^1^O_2_	[120]
CoFe_2_O_4_/BC+PAA	Tetracycline hydrochloride	IC^a^ = 10 mg/L; PDS dose = 0.6 mM; catalyst dosage = 1 g/L; pH = 5	96%	^1^O_2_	[121]
FeS/BC+PS	Tetracycline	IC^a^ = 200 mg/L; PMS dose = 10 mM; catalyst dosage = 0.3 g/L; pH = 3.6	87.4%	SO_4_^•−^ and HO^•^	[122]
N-doped/Boehmeria nivea BC+PMS	Tetracycline	IC^a^ = 20 mg/L; PMS dose = 1 mM; catalyst dosage = 0.1 g/L; pH = 7.0	96.5%	^1^O_2_	[123]
N-doped/Magnetic BC+PMS	Sulfadiazine	IC^a^ = 10 mg/L; PMS dose = 1 mM; catalyst dosage = 0.25 g/L; pH = 5.5	95.2%	SO_4_^•−^ and HO^•^	[124]

IC^a^—initial concentration.

**Table 4:** Applications of BC-based catalysts in photocatalytic processes for the remediation of pollutants.

BC-Based Catalytic Systems	Targeted Pollutants	Operational Parameters	Reduction Efficiency	Dominant Radicals	Ref.
TiO_2_/Chitosan BC+UV	Rhodamine B	IC^a^ = 80 mg/L; catalyst dose = 0.5 g/L; power = 500 W; λ < 420 nm; t = 270 min	100%	O_2_^•−^ and HO^•^	[150]
TiO_2_/walnut shells BC+UV	Methyl orange	IC^a^ = 20 mg/L; catalyst dose = 0.25 g/L; power = 500 W; λ = 360 nm; t = 150 min	96.9%	HO^•^	[151]
N-doped/TiO_2_/BC+UV	Methyl orange	IC^a^ = 20 mg/L; catalyst dose = 0.25 g/L; power = 500 W; λ = 360 nm; t = 90 min	97.6%	HO^•^	[152]
g-C_3_N_4_/BC+UV	Enrofloxacin	IC^a^ = 10 mg/L; catalyst dose = 1 g/L; power = 500 W; pH = 6.6, λ < 420 nm; t = 12 h	81.1%	O_2_^•−^ and holes (h^+^)	[153]
g-C_3_N_4_/Crawfish shell BC+visible light	Enrofloxacin	IC^a^ = 10 mg/L; catalyst dose = 1 g/L; power = 500 W; pH = 7, λ > 420 nm; t = 8 h	90%	O_2_^•−^	[154]
g-C_3_N_4_/Graphene-like BC+PMS+visible light	Tetracycline	IC^a^ = 10 mg/L; catalyst dose = 0.2 g/L; PMS dose = 0.2 g/L.; pH = 5.45; t = 60 min	90%	O_2_^•−^ and ^1^O_2_	[155]
K-doped/g-C_3_N_4_/BC+visible light	Naphthalen	IC^a^ = 20 mg/L; catalyst dose = 0.5 g/L; power = 200 W; λ = 400~800 nm; t = 180 min	82.2%	HO^•^, O_2_^•−^ and h^+^	[156]
S-doped/g-C_3_N_4_/BC+visible light	Tetracycline	IC^a^ = 10 mg/L; catalyst dose = 1 g/L; λ > 420 nm; t = 60 min	81.7%	O_2_^•−^ and h^+^	[157]
Fe/Cu/Sludge BC+PI+UV	Diclofenac sodium	IC^a^ = 20 mg/L; PI dose = 5 mM; catalyst dose = 0.1 g/L; power = 60 W; pH = 6.9; λ = 254 nm; t = 60 min	99.7%	IO_3_^•^	[143]
Zn-Co-LDH/BC+UV	gemifloxacin Gntibiotic	IC^a^ = 15 mg/L; catalyst dose = 0.75 g/L; power = 10 W; pH = 5.5; t = 130 min	92.7%	HO^•^	[158]
g-MoS_2_/Straw BC+visible light	Tetracycline hydrochloride	IC^a^ = 20 mg/L; catalyst dose = 10 mg/L; power = 300 W; pH = 5; λ > 420 nm; t = 60 min	90%	h^+^ and HO^•^	[159]
BiOBr/Lignin-BC+visible light	Rhodamine B	IC^a^ = 30 mg/L; catalyst dose = 0.2 g/L; power = 300 W; λ > 420 nm; t = 60 min	99.2%	O_2_^•−^ and h^+^	[160]
Bi_2_O_2_CO_3_/Rice husk BC+visible light	Tetracycline	IC^a^ = 70 mg/L; catalyst dose = 0.6 g/L; power = 300 W, pH = 6.37; t = 60 min	84.7%	^1^O_2_, O_2_^•−^ and h^+^	[161]
SnS_2_/Tea leaves BC+LED light	Amoxi cillin	IC^a^ = 20 mg/L; catalyst dose = 0.2 g/L; power = 23 W; pH = 5; t = 120 min	93.7%	HO^•^	[57]

IC^a^—initial concentration.

**Table 5:** Applications of BC-based catalysts in sonocatalytic processes for the remediation of pollutants.

BC-Based Catalytic Systems	Targeted Pollutants	Operational Parameters	Reduction Efficiency	Dominant Radicals	Ref.
Rice husk BC	Propylparaben	IC^a^ = 1 mg/L; catalyst dose = 125 mg/L; pH = 5.7, t = 45 min; power = 36 W	78%	HO^•^	[183]
Chitosan/TiO_2_/Grapefruit BC	Ciprofloxacin	IC^a^ = 10 mg/L; catalyst dose = 1 g/L; pH = 6.0, t = 25 min; power = 150 W	85.23%	O_2_^•−^, h^+^ and HO^•^	[186]
ZnO nanorods/Wheat husks and paper sludge BC	Gemifloxacin	IC^a^ = 20 mg/L; catalyst dose = 1.5 g/L; pH = 5.5, t = 45 min; power = 300 W	96.10%	HO^•^	[187]
Fe-Cu-LDH/BC	Cefazolin sodium	IC^a^ = 0.1 mM; catalyst dose = 1 g/L; pH = 6.5, t = 80 min; power = 300 W	97.60%	HO^•^	[188]
Ag_3_PO_4_–Fe_3_O_4_/Bamboo BC	Bisphenol A	IC^a^ = 10 mg/L; catalyst dose = 50 mg/L; pH = 7, t = 60 min; power = 177 W	100%	HO^•^	[189]

IC^a^—initial concentration.

**Table 6:** Literature studies on BC-based catalysts in electrocatalytic systems for pollutant degradation.

BC-Based Catalytic Systems	Targeted Pollutants	Operational Parameters	Current/ Potential	Reduction Efficiency	Dominant Radicals	Ref.
Sludge BC	Methyl orange	IC^a^ = 10 mg/L; t = 240 min; Na_2_SO_4_ dose = 0.1 mM; d = 2 cm	0.05 A	94.5%	HO^•^	[205]
Sludge BC+PMS	Imidacloprid	IC^a^ = 0.5 mg/L; pH = 3.0, t = 60 min; PMS dose = 1 M, catalyst dose = 0.2 g/L	25 V	95.%	HO^•^ and ^1^O_2_	[206]
Lignin-derived BC	Dexamethasone	IC^a^ = 25 mg/L; pH = 3, t = 120 min; Na_2_SO_4_ dose = 50 mM; d = 2.0 cm	11.11 mA/cm^2^	100%	HO^•^	[207]
Banana-peel BC	Bromophenol blue	IC^a^ = 10 mg/L; pH = 7, t = 60 min; Na_2_SO_4_ dose = 10 mM; d = 3.0 cm	100 mA	87.4%	/	[208]
Banana-peel BC+PS	Ibuprofen	IC^a^ = 2 mg/L; pH = 3, t = 120 min; Na_2_SO_4_ dose = 3 mM; d = 3.0 cm	250 mA	100%	HO^•^ and SO_4_^•−^	[209]
Nickel foam/Sludge BC+PS	Sulfamethazine	IC^a^ = 50 mg/L; pH = 3.0, t = 60 min; PS dose = 10 mM; Na_2_SO_4_ dose = 40 mM; d = 2.0 cm	50 mA	100%	HO^•^ and SO_4_^•−^	[210]
ZnCl_2_/BC	Phenol	IC^a^ = 40 mg/L; pH = 3.0, t = 90 min; Na_2_SO_4_ dose = 0.1 M; d = 3 cm	−0.25 V	100%	HO^•^	[211]
PTFE/Black carbon+PMS	Carbamazepine	IC^a^ = 0.042 mM; pH = 3.0, t = 40 min; PMS dose = 50 mM; Na_2_SO_4_ dose = 0.1 M	0.0286 A/cm^2^	97.6%	HO^•^, SO_4_^•−^, ^1^O_2_, and O_2_^•−^	[196]
Ti-Sn-Ce/Bamboo BC	Coking wastewater	IC^a^ = 2 mg/L; pH = 7.9, t = 150 min; Na_2_SO_4_ dose = 3 mM; d = 2.0 cm	30 mA/cm^2^	100%	HO^•^	[212]
N-doped/Kapok fiber BC	Sulfamethoxazole	IC^a^ = 10 mg/L; pH = 5, t = 60 min; Na_2_SO_4_ dose = 50 mM	11 mA/cm^2^	100%	HO^•^	[213]
N-doped/Coffee residues BC	Tetracycline	IC^a^ = 100 mg/L; pH = 7, t = 120 min; Na_2_SO_4_ dose = 5 mM; d = 2.0 cm	4 mA/cm^2^	70.4%	HO^•^, ^1^O_2_, and O_2_^•−^	[214]
Fe/N-doped/BC catalytic membrane	Tetracycline	IC^a^ = 5 mg/L; pH = 7, t = 180 min; Na_2_SO_4_ dose = 20 mM	0.01 mA/cm^2^	100%	HO^•^ and O_2_^•-^	[215]

IC^a^—initial concentration.

## Data Availability

No new data were created or analyzed in this study.
